# The Risk Factors of Nonalcoholic Fatty Liver Disease in Morbidly Obese Patients Undergoing Bariatric Surgery in Iran

**DOI:** 10.1155/2022/5980390

**Published:** 2022-02-07

**Authors:** Ladan Aghakhani, Neda Haghighat, Masoud Amini, Seyed Vahid Hosseini, Seyed Jalil Masoumi

**Affiliations:** ^1^Student Research Committee, School of Nutrition and Food Sciences, Shiraz University of Medical Sciences, Shiraz, Iran; ^2^Laparoscopy Research Center, School of Medicine, Shiraz University of Medical Sciences, Shiraz, Iran; ^3^Laparoscopy Research Center, Department of Bariatric Surgery, Shiraz University of Medical Sciences, Shiraz, Iran; ^4^Department of Surgery, Colorectal Research Center, Shiraz University of Medical Sciences, Shiraz, Iran; ^5^Nutrition Research Center, School of Nutrition and Food Sciences, Shiraz University of Medical Sciences, Shiraz, Iran

## Abstract

**Background and Aims:**

Nonalcoholic fatty liver disease (NAFLD) is common in severely obese individuals undergoing bariatric surgery. Assessing the prevalence and severity of NAFLD seems crucial since it may affect the prevention or development of more severe forms of fatty liver.

**Methods:**

This cross-sectional study was conducted on 228 severely obese individuals undergoing bariatric surgery. Abdominal ultrasonography was done, and clinical and biochemical factors (liver enzymes, lipid profile, and fasting blood sugar (FBS)) were assessed.

**Results:**

The mean body mass index (BMI) was 43.45 ± 5.92 kg/m^2^. The prevalence of NAFLD was 49.12% (mild steatosis: 37.5%, moderate steatosis: 36.6%, and severe steatosis: 25.8%). The main risk factors of NAFLD were weight (*p* = 0.002), BMI (*p* = 0.003), alanine aminotransferase (ALT) (*p* < 0.001), aspartate aminotransferase (AST) (*p* < 0.001), serum triglycerides (TGs) (*p* = 0.004), and FBS (*p* = 0.039). The results revealed a statistically significant decrease in the mean level of high-density lipoprotein cholesterol (HDL-C) (*p* = 0.044). However, no significant association was found between the severity of liver steatosis and the presence of comorbidities such as hypertension, diabetes, hypothyroidism, and dyslipidemia.

**Conclusions:**

More severe NAFLD was associated with increased weight and BMI. Elevated ALT, AST, TG, and FBS levels and decreased HDL-C levels were also the risk factors of NAFLD and its progress to more severe conditions.

## 1. Introduction

Nonalcoholic fatty liver disease (NAFLD) refers to excessive liver adiposity with various histological abnormalities, which can be classified into two categories: nonalcoholic fatty liver and nonalcoholic steatohepatitis (NASH). Nonalcoholic fatty liver has been defined as hepatic steatosis without any significant inflammation leading to hepatocellular injury or fibrosis, whereas NASH has been described as hepatic steatosis with inflammation. Given that NASH is a more severe stage of NAFLD, it is more likely to develop advanced cirrhosis, liver failure, and hepatocellular carcinoma [1, 2]. The prevalence of NAFLD increases primarily by the increasing prevalence of obesity, diabetes mellitus (DM), hyperlipidemia, and polycystic ovary syndrome (PCOS) [3]. The global prevalence rate of NAFLD has been estimated as 25% in the general population [4]. In Iran, as a developing country, the prevalence of NAFLD has been reported as 21.5% [3]. Insulin resistance and dyslipidemia have been considered the main pathognomonic factors of the pathogenesis of NAFLD. Moreover, obesity and central obesity have been found to be associated with an increase in free fatty acid supply to the liver followed by insulin resistance [5].

Evidence of hepatic steatosis must be present on imaging or histology to diagnose NAFLD, and other causes of liver disease or steatosis must be excluded. In spite of the fact that liver biopsy is the gold standard for diagnosing and staging NAFLD, there are some limitations such as invasiveness and variability of sampling, causing this method not to be recommended for all patients. In this context, evaluation of hepatic steatosis by abdominal ultrasound has received much attention, because it is simple, inexpensive, easily tolerated, and noninvasive. Furthermore, it can provide valuable information. Generally, hepatic steatosis on abdominal ultrasound can be defined by at least two of the following findings: liver brightness, vascular blurring, increased hepatorenal contrast, and deep attenuation [6–8]. However, considering the increased prevalence of NAFLD when combined with morbid obesity, it is essential to recognize and evaluate the related risk factors and predictors. Therefore, the present study is aimed at estimating the prevalence of NAFLD and determining its most critical risk factors in morbidly obese patients undergoing bariatric surgery.

## 2. Materials and Methods

### 2.1. Patients and Study Design

This cross-sectional study was conducted on 228 patients with severe obesity undergoing bariatric surgery in the Obesity Center of Mother and Child Hospital in Shiraz between June 2020 and June 2021. We calculated the sample size using the prevalence of NAFLD in Asian countries (12-24%) [9] with a power of 80%. The primary outcome of the study was the prevalence of NAFLD, and the secondary outcome was the investigation of the clinical and biochemical risk factors of NAFLD among morbidly obese patients. The study was approved by the Institutional Ethics Committee of Shiraz University of Medical Sciences (IR.SUMS.REC.1399.245). After providing the patients with explanations about the purpose of the study and the confidentiality of their data, their written informed consent was obtained. The inclusion criteria were age > 18 years and body mass index (BMI) ≥ 40 kg/m^2^ or BMI ≥ 35 kg/m^2^ with one or more comorbidities attributable to obesity. The exclusion criteria were alcohol consumption ≥ 20 g per day for females and ≥30 g per day for males, menopause, lactation, hepatotoxic drugs intake, chronic hepatitis B or C virus infection, and other liver diseases.

### 2.2. Diagnosis of NAFLD

Abdominal ultrasound was used as the reference for diagnosing NAFLD among the patients. The same radiologist performed all ultrasound scans using a SonoAce R5 ultrasound device (SonoAce R5 ultrasound system, South Korea) with a convex probe of 3.5 MHz. According to the previously reported diagnostic criteria, ultrasonic hepatic steatosis was defined based on a comparative evaluation of image brightness compared to the kidneys. Fatty liver causes an increase in the echogenicity of the liver parenchyma [10]. Fatty liver grading has been described in Table 1 [11]. Accordingly, the grade of fatty liver was established.

### 2.3. Data Collection

#### 2.3.1. Anthropometric Measurement

Weight was measured using a digital scale (Seca, Hamburg, Germany) to the nearest 0.1 kg in light clothing and without shoes. Height was measured using a wall-mounted stadiometer to the nearest 0.1 cm. BMI was calculated by dividing weight (kg) by height squared (m). Hip circumference was measured at the level of the widest circumference portion of the buttocks. Waist circumference (WC) was also measured in the smallest part of the waist to the nearest 0.1 cm. WC > 40 inches (102 cm) among males and >35 inches (88 cm) among females was considered a risk factor for health problems [12]. Waist-to-hip ratio (WHR) was calculated by dividing WC by hip circumference. WHR > 0.85 in females and >1.00 in males indicated a health risk [12]. Finally, a standardized protocol was used to measure systolic and diastolic blood pressure.

#### 2.3.2. Biochemical Measurement

Biochemical measurements included alanine aminotransferase (ALT), aspartate aminotransferase (AST), alkaline phosphatase, total cholesterol, triglyceride (TG), high-density lipoprotein cholesterol (HDL-C), low-density lipoprotein cholesterol (LDL-C), and fasting blood sugar (FBS) levels. Moreover, type 2 diabetes mellitus (T2DM) was diagnosed based on the fasting plasma glucose concentration ≥ 126 mg/dl, positive history, and being under antidiabetic medications.

### 2.4. Statistical Analysis

The collected data were analyzed using the SPSS software, version 22. The data were presented as mean ± standard deviation (SD) or as absolute values and percentages. Kolmogorov–Smirnov test was used to analyze the normal distribution of the study variables. Then, chi-square test or Fisher's exact test was used to analyze the categorical variables. Univariate analysis of continuous variables was done using independent sample *t*-test and Mann–Whitney *U* test (in case of nonnormal distribution of the variables). Moreover, changes in the clinical and biochemical variables were compared using the analysis of variance (ANOVA) and Kruskal-Wallis nonparametric test, and Bonferroni test was used for pairwise comparisons. *p* < 0.05 was considered statistically significant.

## 3. Results

### 3.1. Baseline Characteristics of the Patients Undergoing Bariatric Surgery

The anthropometric, demographic, and clinical characteristics of the enrolled patients have been presented in Table 2. The participants included 228 patients with the mean age of 37.04 ± 11.28 years. Among the patients, 187 (80%) were female and 41 (18%) were male. The mean weight of the participants was 116.72 ± 19 kg (median: 112.20 and range: 80.40–180), and their mean BMI was 43.45 ± 5.92 kg/m^2^ (median: 42.63 and range: 30.37–63). Moreover, 35 patients (18.5%) had hypertension, 17 (7.8%) had DM, 38 (21.1%) had hypothyroidism, and 78 (36.1%) had dyslipidemia.

### 3.2. Liver Steatosis

Overall, 49.12% of the participants had fatty liver. Mild liver steatosis (grade I) was found in 42 patients (37.5%), moderate liver steatosis (grade II) in 41 patients (36.6%), and severe liver steatosis (grade III) in 29 patients (25.8%).

### 3.3. Risk Factors Associated with NAFLD

Comparison of the characteristics, comorbid conditions, and biochemical factors of the participants with and without NAFLD has been presented in Table 3. The results revealed no significant difference between the two groups in terms of age, WC, and WHR. As for weight and BMI, there was a significant difference between the patients with severe steatosis (grade III) and those without NAFLD (*p* = 0.005). The results also showed no statistically significant difference between the patients with and without NAFLD regarding comorbidities such as hypertension, diabetes, hypothyroidism, and dyslipidemia. Nonetheless, dyslipidemia was more prevalent in the NAFLD group (53.84%).

**Figure 1:**
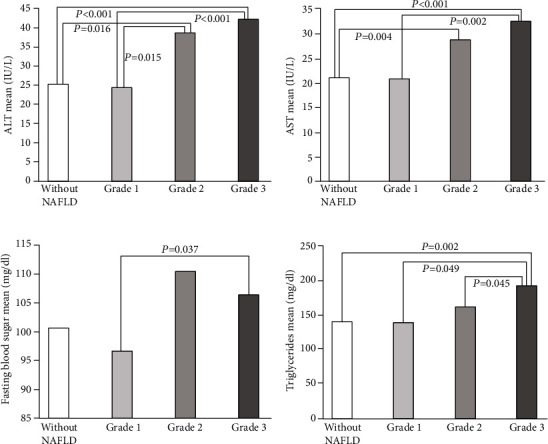
The means of (a) alanine aminotransferase (ALT), (b) aspartate transaminase (AST), (c) fasting blood sugar (FBS), and (d) triglycerides (TGs) in the patients with and without NAFLD undergoing bariatric surgery.

Among the biochemical factors, those with statistically significant differences have been shown in Figure 1. Accordingly, serum ALT levels were higher in the patients with grade II (40.48 ± 28.64 IU/L) and grade III (42.52 ± 20.57 IU/L) liver steatosis compared to those with grade I liver steatosis (23.23 ± 11.25 IU/L) and the group without NAFLD (25.40 ± 15 IU/L). Serum AST levels were also higher in the patients with grade II (28.76 ± 13.98 IU/L) and grade III (32.52 ± 14.88 IU/L) in comparison to those without NAFLD (21.10 ± 10.81 IU/L). There was also a statistically significant difference between the patients with grade I and grade III in this regard (*p* = 0.002). In addition, serum TG levels were higher in the patients with grade III (191.52 ± 95.62 mg/dl) than in those with grade II (161.24 ± 72.04 mg/dl) (*p* = 0.045), those with grade I (137.88 ± 55.40 mg/dl) (*p* = 0.049), and the group without NAFLD (140.50 ± 61.57 mg/dl) (*p* = 0.002). Fasting blood glucose was also higher in patients with grade III (106.35 ± 16.55 mg/dl) compared to those with grade I (96.53 ± 14.59 mg/dl) (*p* = 0.037). Furthermore, a significant decrease was observed in the mean level of HDL-C in the study population (*p* = 0.044).

## 4. Discussion

Obesity-associated NAFLD includes a spectrum of histological abnormalities ranging from steatosis to the inflammatory form of NAFLD, known as NASH. It is frequently seen in severe obesity, and its prevalence has been found to increase up to 90% in such patients [13, 14]. However, little is known about the prevalence and severity of NAFLD among severely obese patients undergoing bariatric surgery in Iran. Therefore, the primary objective of this prospective cohort study was to identify the factors associated with the prevalence and severity of NAFLD in morbidly obese patients undergoing bariatric surgery.

The present study results indicated that the prevalence of NAFLD was 49.12%, and the mean BMI of the patients was 43.45 ± 5.92 kg/m^2^. However, a lower prevalence (16.7%) was reported in the study carried out by Karimi-Sari et al. [15]. On the other hand, some previous studies revealed a higher prevalence of NAFLD [16–18]. It should be noted that the majority of severely obese patients undergoing bariatric surgery are female.

The results of the current research showed no statistically significant association between NAFLD and the presence of comorbidities such as hypertension, diabetes, hypothyroidism, and dyslipidemia. Some previous studies also demonstrated no significant relationships between the increasing degrees of liver damage and hypertension, diabetes, and dyslipidemia [18–21]. However, some investigations have disclosed that diabetes was associated with NASH and advanced fibrosis [20, 22, 23].

The current study findings indicated that the presence and severity of NAFLD were not significantly associated with age, WC, and WHR. However, increasing weight and BMI were significantly associated with NAFLD. Accordingly, patients with BMI > 50 kg/m^2^ (32.3%) were more likely to have severe NAFLD (grade III). Although several studies have emphasized the association between BMI and NAFLD/NASH [16, 19], most studies have shown no significant relationships between increasing BMI and NAFLD prevalence [17, 18, 22].

In the current research, the severity of NAFLD was associated with increasing levels of ALT and AST. Among the patients, 23.1% had elevated levels of ALT (>41 IU/L in males and >37 IU/L in females) and 8.3% had elevated levels of AST (>37 IU/L). Moreover, the majority of patients with higher mean levels of liver enzymes had BMI ≥ 40 kg/m^2^. It was previously established that metabolic syndrome and obesity were associated with elevated liver enzymes, particularly high serum ALT activity. Besides, almost 50% of obese people had elevated ALT levels in addition to NAFLD [24, 25]. In the same vein, Milić et al. conducted a large study on 799 obese patients and concluded that the median ALT and AST levels increased with the obesity class, exceeding the normal limits in 21% of the patients [26].

The present study results revealed a significant relationship between serum HDL-C, TG, and FBS levels and the occurrence of NAFLD, which was in agreement with the results of other studies conducted on the issue [20, 22, 23]. The relationship between obesity and hypertriglyceridemia is possibly mediated by insulin resistance, as studies have suggested that obesity raises the serum TG level as one of the acquired reasons. Moreover, insulin resistance has been recognized as one of the most important pathogeneses of NAFLD. In fact, obesity and hypertriglyceridemia are closely related to insulin resistance, leading to the development and progression of fatty liver [27].

The main limitation of the present study was doing the assessments using ultrasonography, while many previous studies made use of histology to diagnose NAFLD.

This was the first study that reported the status of NAFLD in severely obese Iranian patients undergoing bariatric surgery (sleeve gastrectomy, Roux-en-Y gastric bypass, and single anastomosis sleeve ileal) and analyzed the risk factors of NAFLD in this population.

## 5. Conclusion

In conclusion, the findings demonstrated that more severe NAFLD was associated with increasing weight and BMI, increasing levels of ALT, AST, TG, and FBS, and decreased HDL-C levels. However, hypertension, diabetes, hypothyroidism, and dyslipidemia were not associated with NAFLD and its severity. Detection and evaluation of NAFLD risk factors in severely obese patients undergoing bariatric surgery may effectively prevent NAFLD or its progress to more severe conditions.

## Figures and Tables

**Table 1:** Grading of nonalcoholic fatty liver on ultrasonography.

Mild liver steatosis (grade I)
(i) Least diffuse increase of liver echogenicity
(ii) The liver appears brighter than the kidney cortex
(iii) Standard visualization of intrahepatic vessel walls
Moderate liver steatosis (grade II)
(i) Moderate diffuse increase of liver echogenicity
(ii) A little defective visualization of the intrahepatic vessels
Severe liver steatosis (grade III)
(i) Severe increase of liver echogenicity
(ii) Poor or lack of visualization of intrahepatic vessels
(iii) Poor penetration of the posterior segment of the right lobe of the liver

**Table 2:** Demographic, anthropomorphic, and clinical data of the study population.

No. of patients	228
Age, year	37.04 ± 11.28
Female/male	187 (80)/41(18)
Weight (kg)	116.72 ± 19
Body mass index (kg/m^2^)	43.45 ± 5.92
Waist circumference (cm)	126.47 ± 14.03
Waist-to-hip ratio	0.94 ± 0.09
Obesity classification	
Class 1 (body mass index: 30–34.9 kg/m^2^)	10 (4.4)
Class 2 (body mass index: 35–39.9 kg/m^2^)	50 (22)
Class 3 (body mass index: 40–49.9 kg/m^2^)	136 (59.9)
Super obesity (body mass index > 50 kg/m^2^)	31 (13.7)
Age at the beginning of obesity, no	
Childhood (2-12 years)	86 (47.5)
Teenage (13-17 years)	20 (11)
Youth (18-39 years)	67 (37)
Middle age (40-65 years)	7 (3.9)
Old age (>65 years)	1 (0.6)
Hypertension	35 (18.5)
Type 2 diabetes mellitus	17 (7.8)
Hypothyroidism	38 (21.1)
Dyslipidemia	78 (36.1)

Waist circumference (*n* = 165), waist-to-hip ratio (*n* = 165), age at the beginning of obesity (*n* = 181), hypertension (*n* = 189), type 2 diabetes mellitus (*n* = 217), hypothyroidism (*n* = 180), and dyslipidemia (*n* = 216). The data have been presented as number (%) or mean ± standard deviation.

**Table 3:** Analysis of the clinical and biochemical risk factors of NAFLD.

Risk factors	With NAFLD			Without NAFLD (*n* = 116)	*p* value
	Grade I (*n* = 42)	Grade II (*n* = 41)	Grade III (*n* = 29)		
Age, year	37.14 ± 12.56	37.29 ± 12.85	37.68 ± 10.34	36.76 ± 10.55	0.980
Sex					
Female	39 (92.9)	31 (75.6)	19 (65.5)	98 (84.5)	0.018
Male	3 (7.1)	10 (24.4)	10 (34.5)	18 (15.5)	
Weight (kg)	115.78 ± 15.74	121.69 ± 19.68	126.00 ± 21.92	112.99 ± 18.11	0.002
Body mass index (kg/m^2^)	41.88 ± 5.59	46.64 ± 5.27	45.61 ± 2.82	42.22 ± 5.87	0.003
Waist circumference (cm)	122.90 ± 9.82	131.73 ± 11.93	132.80 ± 12.07	125.84 ± 14.77	0.392
Waist-to-hip ratio	0.92 ± 0.05	0.94 ± 0.05	0.92 ± 0.08	0.94 ± 0.11	0.271
Hypertension (*n*)	4	10	5	16	0.065
Type 2 diabetes mellitus (*n*)	1	3	3	10	0.541
Hypothyroidism (*n*)	5	7	2	24	0.138
Dyslipidemia (*n*)	14	13	15	36	0.306
Alanine aminotransferase (IU/L)	23.23 ± 11.25^a,b^	40.48 ± 28.64^a,c^	42.52 ± 20.57^b,d^	25.40 ± 15.00^c,d^	<0.001
Aspartate aminotransferase (IU/L)	20.76 ± 7.07^a^	28.76 ± 13.98^b^	32.52 ± 14.88^a,c^	21.10 ± 10.81^a,b,c^	<0.001
Alkaline phosphatase (IU/L)	186.73 ± 70.91	196.40 ± 51.04	209.05 ± 49.46	190.73 ± 60.41	0.233
Total cholesterol (mg/dl)	180.26 ± 33.15^a^	184.15 ± 33.82^b^	192.89 ± 40.47^a,b,c^	179.75 ± 35.80^c^	0.347
Triglyceride (mg/dl)	137.88 ± 55.40	161.24 ± 72.04	191.52 ± 95.62	140.50 ± 61.57	0.004
Low-density lipoprotein (mg/dl)	107.75 ± 28.07	116.52 ± 28.65	123.58 ± 38.64	109.10 ± 29.87	0.089
High-density lipoprotein (mg/dl)	46.50 ± 13.01	39.98 ± 11.46	37.05 ± 12.72	44.13 ± 10.89	0.044
Fasting blood sugar (mg/dl)	96.53 ± 14.59^a^	110.48 ± 42.17	106.35 ± 16.55^a^	100.59 ± 26.30	0.039

Waist circumference (*n* = 165), waist to hip (*n* = 165), age at the beginning of obesity (*n* = 181), alanine aminotransferase (*n* = 218), aspartate aminotransferase (*n* = 218), alkaline phosphatase (*n* = 194), total cholesterol (*n* = 211), triglycerides (*n* = 213), low-density lipoprotein (*n* = 206), high-density lipoprotein (*n* = 183), and fasting blood sugar (*n* = 210). The data have been presented as mean ± standard deviation for quantitative variables and number (%) for qualitative ones. ^a,b,c^Significant differences between the groups by the Bonferroni test.

## Data Availability

The data used to support the findings of this study are available from the corresponding author upon request.
